# Sexual Function in Women with and Without PCOS after Metabolic Bariatric Surgery: Insights from a Prospective Cohort Study

**DOI:** 10.1007/s11695-026-08625-1

**Published:** 2026-03-26

**Authors:** Charlotta Klevedal, Yücel Cengiz, Marie Bixo, Sahruh Turkmen

**Affiliations:** 1 Department of Clinical Sciences, Obstetrics and Gynaecology, Umeå University, 90187 Umeå, Sweden; 2 Department of Obstetrics and Gynaecology, Länssjukhuset Sundsvall-Härnösand, 85186 Sundsvall, Sweden; 3 Department of Diagnostics and Intervention, Umeå University, Umeå, Sweden; 4 Department of Surgery, Länssjukhuset Sundsvall-Härnösand, 85186 Sundsvall, Sweden

**Keywords:** Polycystic ovary syndrome, Obesity, McCoy female sexuality questionnaire, Testosterone, Mini international neuropsychiatric interview, Sleeve gastrectomy, Roux-en-Y gastric bypass

## Abstract

**Background:**

Polycystic ovary syndrome (PCOS) and obesity have been linked to impaired sexual function. This study investigated changes in sexual function in women with obesity with and without PCOS after metabolic bariatric surgery.

**Methods:**

We conducted a prospective cohort study of women with obesity who underwent metabolic bariatric surgery, comparing those with (OP) and without (ONP) PCOS. A control group of healthy women without obesity was also included. Sexual function (McCoy Female Sexuality Questionnaire; MFSQ), psychiatric health (Mini International Neuropsychiatric Interview; MINI), menstrual pattern, and testosterone hormone levels were assessed pre-surgery and 12 months post-surgery.

**Results:**

The study included 104 women: 67 with obesity (38 OP, 29 ONP) who underwent metabolic bariatric surgery and 26 healthy controls. Before surgery, both obese groups had lower MFSQ scores (OP: 48 [36.5–54]; ONP: 38 [32–48]) compared to control (52.5 [45.75–56.25]; *p* = 0.001), with no difference between OP and ONP. The OP group had higher testosterone, free androgen index (FAI), hirsutism, and menstrual irregularity (all *p* < 0.001) than other groups, these factors improved post-surgery. MINI scores showed no group differences pre- or post-surgery. After surgery, sexual function improved in both obese groups, aligning with control. Summed MFSQ scores rose significantly only in ONP (*p* = 0.031). Satisfaction with frequency of sexual activity improved in both groups; sexual thoughts improved only in ONP. Lubrication was greater in OP (*p* = 0.019), while orgasm frequency remained lower in both obese groups (*p* = 0.029). In OP, BMI was negatively linked to MFSQ (β = -0.864, *p* = 0.005).

**Conclusions:**

Metabolic bariatric surgery improves sexual function and PCOS symptoms in women with obesity, potentially linked to BMI reduction. Mental health and testosterone levels were not associated with sexual function in this sample, though these null findings should be interpreted cautiously.

**Graphical Abstract:**

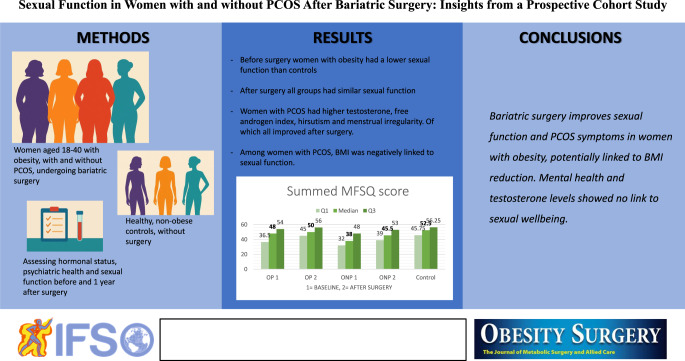

## Key Points

Before surgery, women with obesity had impaired sexual function compared with controls.

After surgery all groups had similar sexual function.

Among women with PCOS, BMI was negatively linked to sexual function.

## Introduction

Polycystic ovary syndrome (PCOS) is a hormonal disorder that affects women of reproductive age, with a prevalence of around 5–16% in the European population [1]. The condition can lead to a variety of symptoms and health problems, including cardiovascular diseases, irregular periods, and infertility.

Previous studies have shown that PCOS can impair sexual function in various ways, including lower desire, frequency of orgasm, and satisfaction [2–4]. However, elevated androgen levels in women with PCOS may contribute to diminished sexual function, while paradoxically enhancing orgasm frequency and sexual interest [2, 4–6]. The physical manifestations of PCOS, such as hirsutism, acne, and weight gain, can affect a woman’s body image and self-esteem, and are connected to impaired sexual function [3, 7, 8]. The genetic foundation, persistent nature, and physical and emotional symptoms of PCOS, have been associated with heightened levels of stress, anxiety, and depression [8, 9], and the connection between PCOS and mental health is well established [2, 7]. These psychological factors can significantly impact a woman’s interest in, and enjoyment of, sexual activity [2, 8]. 

Obesity is strongly associated with PCOS, and it is estimated that > 60% of women with PCOS have overweight or obesity [10]. There is also evidence linking obesity to impaired sexual function [11, 12], and some argue that excessive weight, rather than PCOS, leads to impaired sexual function [11, 13]. However, others find a strong connection between sexual dysfunction and PCOS in women with the same body mass index (BMI) [4]. Thus, conflicting conclusions exist regarding the underlying factors [14]. 

Metabolic bariatric surgery is an effective method of treating women with obesity with PCOS [15], and has been shown to significantly reduce weight and improve metabolic status [15–17]. Furthermore, it affects hormonal status through decreased levels of testosterone and a lower free androgen index (FAI), and reduces prevalence of hirsutism, irregular menstruation, and infertility [15, 16, 18]. This leads to our key question: if all of the aforementioned issues improve after metabolic bariatric surgery, then how does it impact sexual function?

This study had two related objectives. First, we aimed to investigate the independent contributions of obesity and PCOS to sexual function by comparing women with obesity, with and without PCOS, to a healthy non-obese control group at baseline. Second, we sought to evaluate the impact of metabolic bariatric surgery on sexual function in women with obesity, both with and without PCOS, by assessing changes 12 months postoperatively. This dual approach allows us to interpret postoperative improvements in sexual function within the context of baseline differences related to obesity and PCOS.

## Methods

### Study Population

The participants were recruited from a cohort of women with obesity who qualified for metabolic bariatric surgery at the Department of Surgery at the Sundsvall County Hospital in Sweden between 2009 and 2024. The patient group was divided into two groups based on PCOS diagnosis: women with obesity diagnosed with PCOS (the OP group), and women with obesity not diagnosed with PCOS (the ONP group). Women were considered to have PCOS based on the Rotterdam 2003 criteria, i.e. at least two of the following three: (1) polycystic ovarian morphology on ultrasound, (2) clinical and/or biochemical hyperandrogenism (Ferriman-Gallwey score ≥ 8 and/or FAI ≥ 5, and 3) oligo- or amenorrhoea. In addition, other relevant disorders were excluded [19]. A third group of participants, consisting of healthy women without obesity who had not undergone surgery, was recruited as the control group between 2022 and 2025 through advertisements.

The inclusion criteria for the women with obesity were an age of 18–40 years and a BMI > 35 kg/m². For the control group, the inclusion criteria were women aged 18–40 years with a BMI < 30 kg/m^2^. Across all groups, exclusion criteria included the use of psychoactive medication in the month prior to enrollment, severe current psychiatric conditions that could substantially impair the participant’s ability to think clearly, communicate effectively, or reliably describe their symptoms and experiences, premenstrual dysphoric disorder (PMDD), a history of or ongoing alcohol or drug abuse, significant somatic illness, pregnancy within the preceding three months, and ongoing breastfeeding. Psychiatric assessment was conducted using the Mini-International Neuropsychiatric Interview (MINI) together with clinical evaluation. Psychiatric exclusion criteria were applied only to conditions associated with marked current functional impairment, including current psychotic disorders, acute manic episodes of bipolar disorder, severe major depressive episodes with significant cognitive impairment, and moderate to severe neurocognitive disorders. Lifetime psychiatric diagnoses without current functional impairment were not exclusionary.

Importantly, while patient groups were actively seeking care for specific medical issues, controls were recruited via advertisements and were not seeking medical or psychiatric care. Thus, the control group was not inherently enriched for psychiatric conditions or distress, although some positive findings were observed.

### Study Design

A prospective cohort study was conducted among women with obesity who underwent laparoscopic metabolic bariatric surgery, either sleeve gastrectomy (SG) or Roux-en-Y gastric bypass surgery (GBP). The initial assessment of the patient groups took place at the Department of Surgery at Sundsvall County Hospital, Sweden. Patients were provided with detailed information about the planned surgery, including expected outcomes and potential risks. They also received oral and written information about the study, and signed consent forms. Following this, BMI calculations were performed, and blood samples were collected to analyse total testosterone and sex hormone-binding globulin (SHBG).

The patients then proceeded to the Department of Obstetrics and Gynaecology for further examination. Information about previous pregnancies, deliveries, menstrual patterns, contraception use, and partner status was collected. The patients underwent a gynecological examination, which involved evaluating the effects of high androgen hormones (body-hair growth) using the modified Ferriman-Gallwey (MFG) scoring system and checking the PCOS diagnostic criteria. Additionally, patients participated in a Mini International Neuropsychiatric Interview (MINI), and completed the McCoy Female Sexuality Questionnaire (MFSQ).

The women with obesity in the patient groups underwent metabolic bariatric surgery (SG or GBP) following inclusion, and subsequently received postoperative care at the Department of Surgery. The control group did not undergo surgery but was assessed for the same study parameters; as such, blood samples, physical measurements, and questionnaires were completed, but only on one occasion.

The degree of hair growth is typically quantified using the MFG scoring system, wherein terminal hair growth is scored from 0 to 4 at nine anatomical sites, and these scores are summed (maximum score 36) [20, 21]. Different MFG score thresholds have been proposed to diagnose hyperandrogenism, ranging from ≥ 3 to ≥ 8. The most optimal threshold to use remains in question; the most recent guideline recommend a threshold of ≥ 4 to 6, depending on ethnicity, but this may result in the overdiagnosis of the condition as compared to the traditionally used threshold of ≥ 8 [22]. In this study, a score of ≥ 8 was considered the cutoff value, as it is commonly used in European populations [23, 24]. 

Twelve months after surgery, the patients were re-examined for the same parameters; blood samples were collected, and the MFSQ and MINI were completed at the Department of Obstetrics and Gynaecology at Sundsvall County Hospital. Information about previous pregnancies, deliveries, menstrual patterns, contraception use, and partner status was collected. Venous blood samples were taken to measure testosterone and SHBG. The degree of hair growth was assessed using the MFG scoring system.

The MINI is a structured diagnostic tool designed to assess a variety of psychiatric disorders quickly and effectively. It aligns with the diagnostic criteria of the Diagnostic and Statistical Manual of Mental Disorders (DSM) and International Classification of Diseases (ICD) [25]. The MINI has been used in Swedish clinical settings [26], and been shown to have a high sensitivity and specificity [27]. The MINI evaluates the following psychiatric conditions: major depressive disorder (current, recurrent, and lifetime), suicidality, bipolar disease (current and lifetime), panic disorder (current and lifetime), agoraphobia, social phobia, obsessive-compulsive disorder, post-traumatic stress disorder, generalized anxiety disorder, alcohol dependence and abuse, drug dependence and abuse, psychotic disorders, anorexia nervosa, bulimia, generalized somatic symptom disorder, and antisocial personality disorder. It employs standardized screening questions, with additional follow-up questions if a positive response is given. This structured approach ensures a thorough and systematic assessment, making it widely used in both clinical and research contexts for its reliability and practicality.

The MFSQ was originally developed to assess sexual function in postmenopausal women [28], and later revised and validated for broader use [29]. It has also been used to investigate sexual function in women with PCOS [2, 11, 13]. The questionnaire includes multiple items that assess sexual desire, frequency of sexual activity, satisfaction, and other related experiences. The first 9 (of 12) items are measured on a 7-point Likert scale and are summed, with Items 6 and 7 inverted so that a higher score always corresponds to better sexual function, resulting in a score range of 9–63. This summed score of Items 1–9, rather than the total for all 12 items, is most commonly used. The summed score for the MFSQ represents an overall measure of various aspects of female sexual function and satisfaction, and provides a comprehensive overview of an individual’s sexual function for research or clinical purposes. To ensure comparability with other studies, we used the Swedish version of the MFSQ with 9 items, supplemented by three additional questions, focusing on the widely adopted 9-item score [2, 30]. 

### Hormonal Analyses

Serum or plasma levels of testosterone (nmol/l) and SHBG (nmol/l) were measured using an electrochemiluminescence immunoassay (ECLIA) with the cobas e601 and e801 analytical units (Roche Diagnostics GmbH, Mannheim, Germany) at the medical laboratory of Sundsvall County Hospital, which is certified by the Swedish Board for Accreditation and Conformity Assessment. The intra-assay variations for both measurements were 4%, while the inter-assay variations were 2%. Between 2016 and 2018 there was a shift in the laboratory method, from samples of serum to plasma. For testosterone, the measuring range was 0.42 or 0.5 nmol/l to 52.0 nmol/l. For levels below this, the output reported < 0.42 nmol/l or < 0.5 nmol/l, depending on the method of analysis. For SHBG, the measuring range was 0.8–200 nmol/l. For levels above the detectable range, the output reported > 200 nmol/l. In cases where values were below or above this, the measuring range values were set to the corresponding limit value, i.e. < 0.42 to 0.42, < 0.5 to 0.5, and > 200 to 200, in order to compute an FAI value (testosterone/SHBG × 100) for all participants.

### Statistical Analysis

Data were analysed using IBM SPSS Statistics for Windows, Version 29.0 (Armonk, NY: IBM Corp). Tests for skewness showed that the data were not normally distributed; thus, non-parametric tests were used. The primary statistical analysis compared outcome parameters among three groups – women with obesity with PCOS, women with obesity without PCOS, and healthy women without obesity as the control group – at baseline and, for the patient groups, after metabolic bariatric surgery. Pearson’s χ² test was used for dichotomous variables, and the Kruskal-Wallis test for continuous variables. The Mann-Whitney U test was utilized to compare the GBP and SG groups, and the Wilcoxon test was used to detect changes in study parameters due to metabolic bariatric surgery within groups. Univariate and multivariate regression analyses were conducted to investigate associations between outcome parameters. Simple linear regression was performed to select candidate variables for multivariable linear regression. All variables with p-values < 0.05 during the simple linear regression were considered as candidate variables for the multivariable linear regression. After the multivariable linear regression analysis, variables with p-values < 0.05 were considered to have a statistically significant association with the dependent variable. The strength of association between independent and dependent variables was assessed using unstandardized β with 95% confidence interval (CI). Data for continuous variables are presented as medians and interquartile ranges (IQR), and nominal variables are presented as percentages (%) and number of participants (n). The significance level was set at *p* < 0.05.

#### Power Analysis and Sample Size

Power calculations were based on previously published data on sexual dysfunction in women with obesity undergoing. Kinzl et al. reported that 45% of morbidly obese women experienced sexual dysfunction before gastric banding, decreasing to 12% after surgery, corresponding to an absolute reduction of approximately 33% points in the prevalence of sexual dysfunction [31]. Assuming a similar magnitude of improvement in our population of women with obesity and PCOS, we performed a power calculation for a within‑group comparison of the proportion of women with sexual dysfunction before and after surgery (paired binary outcome). With an alpha level of 0.05 (two‑sided), a power of 80%, and an expected reduction from 45% to 12%, the required sample size for detecting this change in the PCOS group was estimated to be 14 women. This value corresponds to the minimum number of women with PCOS needed to detect a large, clinically meaningful improvement in sexual function comparable to that reported by Kinzl et al.

However, our study was also designed to explore differences between women with and without PCOS and to examine continuous sexual function scores (total MFSQ) rather than only dichotomous dysfunction status. For these between‑group and continuous outcomes, the detectable effect size with our achieved sample (~ 24 women per subgroup at follow‑up) is in the moderate‑to‑large range, and the study is likely underpowered to detect small effects. Rank-based effect sizes (r-values) were calculated for the primary outcome comparisons involving continuous variables to indicate whether the observed differences reflected small effects, no meaningful effects, or imprecisely estimated effects.

## Results

**Fig. 1:**
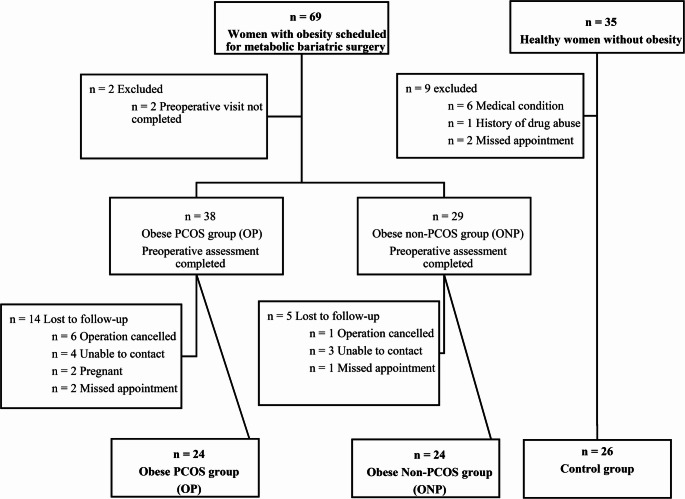
Recruitment flowchart for the study population. SG, sleeve gastrectomy; GBP, Roux-en-Y gastric bypass; PCOS, polycystic ovary syndrome

A total of 104 patients participated in the study. Of the patients in the obese groups, 67 completed preoperative assessments, with 38 classified as OP and 29 as ONP. Postoperative follow-ups were incomplete for 14 OP and 5 ONP patients, leaving 24 patients in each group. The control group initially included 35 women; after applying exclusion criteria and excluding two participants who missed appointments, 26 were eligible (Fig. 1).

During the study period, changes in the operational procedures at the Department of Surgery led to a modification to the metabolic bariatric surgery technique. Because of this, 13 OP patients underwent GBP while the rest, across both groups, underwent SG. We performed a sensitivity analysis where we examined whether the main findings were influenced by surgical methods by re-analysing outcomes separately for patients who underwent GBP and those who underwent SG. Across all these analyses, the results were consistent with the overall findings, indicating that the primary conclusions regarding BMI, MFSQ, and MINI outcomes were not driven by procedure type. Comparing patients with obesity by surgery type revealed higher testosterone levels (nmol/l) in GBP patients (2.2 [1.5]) versus SG patients (1.11 [0.86], *p* = 0.003), consistent with all GBP patients having PCOS. No other significant differences emerged when the patients were analysed by surgery type.

To assess potential attrition bias, baseline characteristics were compared between women with obesity and PCOS and women with obesity without PCOS, separately among participants who completed the 12-month follow-up and those lost to follow-up. No statistically significant differences were observed in age, baseline BMI, total MFSQ score, MINI diagnosis, testosterone, free androgen index (FAI), or relationship status (all *p* > 0.05).

### Baseline

A comparison of baseline characteristics across the three patient groups revealed no significant differences in age, parity, partner status, or MINI test results (Table 1).

**Table 1:** Baseline characteristics of the study groups

	OP	ONP	Control	*P*-value	*P*-value pairwise
Age (years; median; IQR)	27.0 (5.25)	29.0 (7.5)	29.5 (7.25)	0.262	a = 0.432 b = 0.103 c = 0.412
BMI (kg/m^2^; median; IQR)	44.55 (5.48)	42.7 (6.65)	23.6 (3.98)	**< 0.001**	a = 0.406 **b < 0.001 c < 0.001**
Testosterone (nmol/L; median; IQR)	1.70 (0.90)	0.96 (0.51)	1.00 (0.83)	**< 0.001**	**a < 0.001 b < 0.001** c = 0.898
FAI (median; IQR)	9.1 (6.32)	3.42 (3.08)	1.55 (1.86)	**< 0.001**	**a < 0.001 b < 0.001 c = 0.006**
Hirsute					
Yes (%; n)	65.8 (25)	17.2 (5)	11.5 (3)	**< 0.001**	**a < 0.001 b < 0.001** c = 0.549
No (%; n)	34.2 (13)	82.8 (24)	88.5 (23)		
Has a partner					
Yes (%; n)	81.6 (31)	72.4 (21)	88.5 (23)	0.317	a = 0.373 b = 0.456 c = 0.137
No (%; n)	18.4 (7)	27.6 (8)	11.5 (3)		
Has at least one child					
Yes (%; n)	41.2 (14)	51.7 (15)	34.6 (9)	0.429	a = 0.402 b = 0.604 c = 0.201
No (%; n)	58.8 (20)	48.3 (14)	65.4 (17)		
Has regular menstruation (no contraception)					
Yes (%; n)	19.2 (5)	87.5 (14)	100.0 (12)	**< 0.001**	**a < 0.001 b < 0.001** c = 0.204
No (%; n)	80.8 (21)	12.5 (2)	0.0 (0)		
Uses contraception					
Yes (%; n)	18.8 (6)	41.4 (12)	53.8 (14)	**0.018**	a = 0.053 **b = 0.005** c = 0.355
No (%; n)	81.3 (26)	58.6 (17)	46.2 (12)		
MINI					
Positive (%; n)	43.2 (16)	35.7 (10)	42.3 (11)	0.812	a = 0.540 b = 0.941 c = 0.619
Negative (%; n)	56.8 (21)	64.3 (18)	57.7 (15)		

*P*-value: a = OP vs. ONP; b = OP vs. control; c = ONP vs. control. OP = Obese PCOS group. ONP = Obese non-PCOS group. IQR = interquartile range. BMI = body mass index. FAI = free androgen index, testosterone/SHBG x 100. MINI = Mini International Neuropsychiatric Interview

In the control group, 11 women (42.3%) met the diagnostic criteria for at least one disorder according to the MINI test. It is important to note that an individual may fulfil the criteria for multiple diagnoses simultaneously. Among these 11 women, 8 had depressive disorders (current, recurrent, or past episodes of depression), 7 had anxiety disorders (current or past panic disorder, agoraphobia, social phobia, or generalized anxiety disorder), 1 had suicidality, 1 had alcohol abuse, and 1 had a psychotic disorder. In the OP group, 16 women (43.3%) had MINI results suggesting a psychiatric disorder; among them, 9 had depressive disorders, 8 had anxiety disorders, 5 had bipolar disorders (experiencing a hypomanic episode, or a past manic episode), and 1 had alcohol abuse. In the ONP group, 10 women (35.7%) had MINI results suggesting a psychiatric disorder; of these, 9 had depressive disorders, 9 had anxiety disorders, 2 had bipolar disorders, 1 had obsessive-compulsive disorder, and 1 had antisocial personality disorder.

BMI was similar in the OP and ONP groups (44.55 [5.48] vs. 42.7 [6.65], *p* = 0.406, *r* = 0.14) but lower in the control group (23.6 [3.98], *p* < 0.001) with a large effect size (control vs. OP: *r* = 0.85, control vs. ONP: *r* = 0.84). Contraception use rates differed: 18.8% (OP), 41.4% (ONP), and 53.8% (control) (OP vs. ONP, *p* = 0.053; OP vs. control, *p* = 0.005; ONP vs. control, *p* = 0.355). Among non-users, regular menstruation rates were 19.2% (OP), 87.5% (ONP), and 100% (control) (OP vs. ONP, *p* < 0.001; OP vs. control, *p* < 0.001; ONP vs. control, *p* = 0.204) (Table 1).

During the preoperative assessment, the OP and ONP groups had lower summed MFSQ scores than the control group (48 [17.5], 38 [16], 52.5 [10.5], respectively, overall *p* = 0.001), with no significant difference between OP and ONP (*p* = 0.070) with a small effect size (*r* = 0.23). Similar trends were seen for Item 1 (Satisfaction with frequency of sexual activity): 4 [2] (OP), 4 [4] (ONP), 6 [2] (control) (*p* = 0.006), and Item 5 (Frequency of orgasm): 5 [3.5] (OP), 4 [3.5] (ONP), 6 [2] (control) (*p* = 0.007). ONP scored lower than control in Item 2 (Sexual thoughts): 3 [2] vs. 3.5 [2] (*p* = 0.032), Item 3 (Enjoyment): 5 [2.75] vs. 6 [1] (*p* = 0.010), Item 4 (Arousal): 4 [4] vs. 6 [1] (*p* = 0.003), and Item 10 (Frequency of intercourse): 3 [2.5] vs. 3 [3.25] (*p* = 0.028). OP outperformed ONP in Item 6 (Insufficient lubrication): 2 [3.5] vs. 4 [4.5] (*p* = 0.012). No significant differences were found for the other items. The full scores are presented in Table 2.

Serum/plasma testosterone levels (nmol/l) were similar in the ONP and control groups (0.96 [0.51] vs. 1.00 [0.83], *p* = 0.898) with a large effect size (*r* = 0.58) but significantly higher in the OP group (1.70 [0.90], *p* < 0.001). Median FAI values indicated hyperandrogenemia in OP (9.1 [6.32]) and elevated androgen levels in ONP (3.42 [3.08]) compared to control (1.55 [1.86]). Group comparisons: OP vs. ONP (*p* < 0.001) with a large effect size (*r* = 0.62), OP vs. control (*p* < 0.001) with a negligible effect, *r* = 0.03), ONP vs. control (*p* = 0.006, *r* = 0.48, moderate to large effect). MFG scores showed significant differences, with hirsutism diagnosed in 65.8% (OP), 17.2% (ONP), and 11.5% (control) of participants. Significant differences were found for OP vs. ONP and OP vs. control (both *p* < 0.001), while ONP vs. control showed no difference (*p* = 0.549) (Table 1).

To identify predictors of the summed MFSQ score, regression analysis was conducted using age, BMI, MINI test results, partner status, nulliparity, menstrual regularity, contraception use, testosterone, FAI, and Ferriman-Gallwey score. In the OP group, BMI was negatively associated with MFSQ score (β = -0.864, 95% CI: -1.447 to -0.282, *p* = 0.005), while having a partner showed a strong positive association (β = 20.554, 95% CI: 12.185 to 28.923, *p* < 0.001). These associations remained significant after adjusting for the other predictors mentioned above, none of which were significant. In the control group, having a partner was also positively associated with MFSQ score (β = 18.391, 95% CI: 12.351 to 24.431, *p* < 0.001). After controlling for age, BMI, testosterone, FAI, hirsutism, nulliparity, menstrual regularity, contraceptive use, and MINI results, no additional significant associations were identified. In the ONP group, no significant associations were found.

### Follow-up

At the 12-month follow-up, BMI had decreased significantly in both the OP (31.3 [6.1], *p* < 0.001, *r* = 0.88 [very large effect]) and ONP groups (30.1 [8], *p* < 0.001, *r* = 0.88 [very large effect]), but remained higher than the control group (overall *p* < 0.001). No BMI difference was found between the OP and ONP groups (*p* = 0.910, *r* = 0.002 [small effect size]). Summed MFSQ scores increased significantly in the ONP group (38 [16] to 45.5 [14], *p* = 0.031, *r* = 0.45 [moderate to large effect]), while in the OP group they showed a non-significant increase (48 [17.5] to 50 [10], *p* = 0.222, *r* = 0.26 [small to moderate effect]). Item 1 (Satisfaction with frequency of sexual activity) improved in both groups (OP: 4 [2] to 5 [2.75], *p* = 0.032; ONP: 4 [4] to 5 [2.75], *p* = 0.046). Item 2 (Sexual thoughts) improved only in the ONP group (3 [2] to 4 [1], *p* = 0.042). No other significant changes were noted. (Table 2).

**Table 2:** Results of the McCoy Female Sexuality Questionnaire

		Baseline					Follow-up					Intragroup change	
		OP	ONP	Control	*P*-value	*P*-value pairwise	OP	ONP	Control	*P*-value	*P*-value pairwise	OP *P*-value	ONP *P*-value
1	Satisfaction with frequency of sexual activity	4 (2)	4 (4)	6 (2)	**0.006**	a = 0.959 **b = 0.004 c = 0.006**	5 (2.75)	5 (2.75)	6 (2)	NS		**0.032**	**0.046**
2	Frequency of sexual thoughts and fantasies	3 (2)	3 (2)	3.5 (2)	NS	a = 0.484 b = 0.110 **c = 0.032**	4 (2)	4 (1)	3.5 (2)	NS		NS	**0.042**
3	Enjoyment of sexual activity	6 (2)	5 (2.75)	6 (1)	**0.031**	a = 0.066 b = 0.351 **c = 0.010**	7 (1)	6 (2.75)	6 (1)	NS		NS	NS
4	Excitement/arousal during sexual activity	6 (3)	4 (4)	6 (1)	**0.014**	a = 0.100 b = 0.135 **c = 0.003**	6 (2)	6 (2.75)	6 (1)	NS		NS	NS
5	Frequency of orgasm	5 (3.5)	4 (3.5)	6 (2)	**0.007**	a = 0.547 **b = 0.011 c = 0.003**	4 (3)	5 (3)	6 (2)	**0.029**	a = 0.815 **b = 0.016 c = 0.031**	NS	NS
6	Insufficient lubrication	2 (3.5)	4 (4.5)	2 (1)	**0.036**	**a = 0.012** b = 0.589 c = 0.072	2 (1)	2 (2)	2 (1)	**0.019**	**a = 0.008 b = 0.032** c = 0.570	NS	NS
7	Painful sexual intercourse	1 (1)	2 (2)	2 (1)	NS		2 (1)	1 (1)	2 (1)	NS		NS	NS
8	Satisfaction with partner as lover	7 (1)	6 (2)	7 (1)	NS		7 (0.75)	7 (1)	7 (1)	NS		NS	NS
9	Satisfaction with partner as friend	7 (1)	7 (1)	7 (0)	NS		7 (1)	7 (0)	7 (0)	NS		NS	NS
10	Frequency of intercourse	3 (2)	3 (2.5)	3 (3.25)	NS	a = 0.193 b = 0.293 **c = 0.028**	4 (4)	3 (3.75)	3 (3.25)	NS		NS	NS
11	Frequency of masturbation	2 (2)	2 (2)	3 (2)	NS		1 (2)	2 (2.75)	3 (2)	NS		NS	NS
12	Avoidance of sexual intercourse due to pain	1 (1)	1 (1)	1 (0.75)	NS		1 (1)	1 (0)	1 (0.75)	NS		NS	NS
	Summed score items 1–9	48 (17.5)	38 (16)	52.5 (10.5)	**0.001**	a = 0.070 **b = 0.035 c < 0.001**	50 (10)	45.5 (14)	52.5 (10.5)	NS		NS	**0.031**

*P*-value: a = OP vs. ONP; b = OP vs. control; c = ONP vs. control. OP= Obese PCOS group. ONP= Obese non-PCOS group. Numbers presented as medians (interquartile range)

A comparison of the MFSQ scores showed no differences in the summed scores between OP, ONP, *r* = 0.20 (small effect), and control groups. Differences were observed only in Item 5 (Frequency of orgasm) and Item 6 (Insufficient lubrication). Item 5 scores were lower in OP and ONP compared to control (OP: 4 [3], ONP: 5 [3], control: 6 [2]; OP vs. ONP, *p* = 0.815; OP vs. control, *p* = 0.016; ONP vs. control, *p* = 0.031). Item 6 outcomes were better in OP compared to ONP and control (OP: 2 [1], ONP: 2 [2], control: 2 [1]; OP vs. ONP, *p* = 0.008; OP vs. control, *p* = 0.032; ONP vs. control, *p* = 0.570).

Apart from BMI, the only significant difference between the groups was the proportion of women with a high MFG score (OP vs. ONP, *p* = 0.006; OP vs. control, *p* = 0.024; ONP vs. control, *p* = 0.408). In the OP group, PCOS symptoms improved. Serum testosterone levels and FAI normalised in both OP and ONP, with no significant differences at follow-up. High MFG scores decreased to 7 (41.2%) for OP and 1 (4.8%) for ONP, although these changes were not statistically significant. Menstrual patterns normalised across all groups, with regular menstruation observed at follow-up. No significant differences were found between groups for the MINI test results, relationship status, nor contraception use (Table 3).

**Table 3:** Postoperative characteristics and intragroup changes

				Across groups		Intragroup change	
	OP	ONP	Control	*P*-value	*P*-value pairwise	OP *P*-value	ONP *P*-value
BMI (kg/m^2^; median; IQR)	31.3 (6.1)	30.1 (8)	23.6 (3.98)	**< 0.001**	a = 0.910 **b < 0.001 c < 0.001**	**< 0.001**	**< 0.001**
Testosterone (nmol/L; median; IQR)	1.19 (1.08)	0.85 (0.82)	1.00 (0.83)	0.674	a = 0.376 b = 0.560 c = 0.719	**0.003**	0.129
FAI (median; IQR)	1.52 (2.05)	1.43 (2.48)	1.55 (1.86)	0.568	a = 0.713 b = 0.306 c = 0.488	**0.006**	**< 0.001**
Hirsutism							
Yes (%; n)	41.2 (7)	4.8 (1)	11.5 (3)	**0.008**	**a = 0.006 b = 0.024** c = 0.408	0.125	0.5
No (%; n)	58.8 (10)	95.2 (20)	88.5 (23)				
Has a partner							
Yes (%; n)	87.0 (20)	83.3 (20)	88.5 (23)	0.865	a = 0.727 b = 0.873 c = 0.602	¤	¤
No (%; n)	13.0 (3)	16.7 (4)	11.5 (3)				
Has regular menstruation (no contraception)							
Yes (%; n)	100.0 (15)	100.0 (11)	100.0 (12)	*		**0.004**	*
No (%; n)	0.0 (0)	0.0 (0)	0.0 (0)				
Uses contraception							
Yes (%; n)	21.1 (4)	52.2 (12)	53.8 (14)	0.058	**a = 0.039 b = 0.027** c = 0.907	¤	¤
No (%; n)	78.9 (15)	47.8 (11)	46.2 (12)				
MINI							
Positive (%; n)	33.3 (8)	50.0 (12)	42.3 (11)	0.504	a = 0.242 b = 0.514 c = 0.586	0.754	0.219
Negative (%; n)	66.7 (16)	50.0 (12)	57.7 (15)				

*P*-value: a = OP vs. ONP; b = OP vs. control; c = ONP vs. control. OP = Obese PCOS group. ONP = Obese non-PCOS group. IQR = interquartile range. BMI = body mass index. FAI = free androgen index, testosterone/SHBG x 100. MINI = Mini International Neuropsychiatric Interview.* = not possible to compute. ¤ = not computed

The results of the MINI test did not differ between the three groups one year after surgery. However, for OP the proportion of women with MINI test results suggesting a psychiatric disorder decreased by 23%, leaving 8 women with positive results. Among them, 2 had depressive disorders, 8 had anxiety disorders, 3 had bipolar disorders, and 1 had antisocial personality disorder. In the ONP group, in contrast, the number of women with MINI test results suggesting a psychiatric disorder increased by 2, reaching a total of 12 (50%): 9 had depressive disorders, and 8 had anxiety disorders.

To examine whether surgical procedure influenced the primary outcomes, patients were divided into two groups based on operation type (GBP and SG). Statistical comparisons were performed for MFSQ scores, BMI, and MINI outcomes at both baseline and follow-up. No significant differences were observed between the two procedure groups for any of these outcomes at either time point, and no differential change over time was detected.

## Discussion

This study examined sexual function in women with obesity who underwent metabolic bariatric surgery, focusing on its impact on sexual function and the parameters influencing it. Preoperatively, the summed MFSQ score, which reflects an individual’s overall sexual function, was lower in women with obesity, both with and without PCOS, compared to controls without obesity. One year after surgery, no significant differences in summed MFSQ scores were observed among the three groups (OP, ONP, and control). However, within-group analysis revealed a significant improvement in the summed MFSQ score for the ONP group, but not for the OP group.

Both the OP and ONP groups showed significant reductions in BMI, although both remained higher than that of the control group. No significant differences in BMI were noted between the two obese groups. The proportion of women with a high MFG score differed significantly across the groups. In the OP group, symptoms of PCOS improved, with menstrual patterns, testosterone levels, and FAI normalising in both the OP and ONP groups. Furthermore, no significant differences were identified between the groups regarding MINI interview results, relationship status, nor contraception use at 12-month follow-up.

We observed an inverse relationship between BMI and sexual function in women with PCOS. Despite significant postoperative weight reduction in this group, summed MFSQ scores did not show substantial improvement. However, a trend towards improvement was identified, with fewer patients in the lowest quartile, as scores increased from 36.5 to 46. It is possible that other factors, such as the small sample size and/or hormonal levels, may have influenced sexual function and offset the impact of weight loss. Nevertheless, the observed association between BMI and sexual function suggests that weight loss, or achieving a lower weight, could be linked to improvement in sexual function. This finding aligns partially with earlier studies, which also reported improvements in sexual function following metabolic bariatric surgery [32–34]. However, the connection to weight loss remains debated. Treacy et al. found that a minimum weight loss of 13 kg/m² after metabolic bariatric surgery resulted in improved global sexual quality [34]. Conversely, Brunault et al. observed no association between sexual function and the magnitude of weight loss [32]. Differences in study populations, particularly in pre- and postoperative BMI categories, may account for these discrepancies. This suggests that the critical factor may not be the extent of weight loss, but postoperative BMI. Our study supports this, as the participants achieved postoperative BMI values approaching non-obesity.

Hu et al. demonstrated that the likelihood of achieving full remission of PCOS symptoms was strongly associated with postoperative BMI [15]. Similarly, Månsson et al. found that women with PCOS who reported that the condition affected their sex life were more likely to have obesity and have a higher BMI than those without such experiences, who were only slightly overweight [2]. Several factors may explain the association between a lower BMI and improved sexual function: improved body image and self-esteem may boost sexual interest [33], desire, and arousal [35], while a lighter body may better facilitate sexual activity [31, 36]. In our study, although not significant, both obese groups showed a postoperative increase in the frequency of intercourse, excitement during sexual activity, and enjoyment of sexual activity. Additionally, there was a significant increase in satisfaction with the frequency of sexual activity, which we hypothesize may be attributed to the lower BMI.

There was also a connection between the summed MFSQ score and having a partner. This result was expected due to the design of the MFSQ, in that Items 8 and 9 are only scored if the participant has a partner. Additionally, having a partner may facilitate access to sexual activity, potentially influencing Item 10 (Frequency of intercourse) and Item 1 (Satisfaction with the frequency of sexual activity). Other studies have also used the MFSQ in a similar way, accounting for the proportion of women with a partner [2, 37]. Notably, in our study the proportion of women with a partner was comparable across the groups at both baseline and follow-up, thereby minimizing the potential impact of partner status on the results.

Prior studies have demonstrated a strong association between mental health and both PCOS and obesity [5, 8, 22], as well as an improvement in depressive symptoms following metabolic bariatric surgery [33]. However, in this study no differences in mental-health status were observed between the groups before nor after surgery, nor was there any significant change within groups when comparing pre- and postoperative MINI scores. This may be due to the exclusion of patients with ongoing psychiatric conditions, which resulted in the inclusion of participants with only mild or subclinical symptoms. Interestingly, a relatively high proportion of women across all groups had a positive MINI result. Even in the control group, the MINI tests of a considerable number (42.3%) of women suggested that they had at least one psychiatric diagnosis. Several factors may help explain the unexpectedly high proportion of MINI-identified psychiatric diagnoses in the control group. First, the control group was defined only by the absence of obesity and PCOS, and no exclusion criteria related to psychiatric history or current psychological symptoms were applied. This allowed the inclusion of individuals with past, mild, or subclinical psychiatric conditions, which the MINI is designed to detect. Second, controls were recruited via advertisements and were not actively seeking medical or psychiatric care, meaning that they were not inherently enriched for psychological distress or other health concerns. Third, the MINI includes modules assessing both current and lifetime diagnoses, which can increase the likelihood of identifying historical or subthreshold conditions. Finally, the MINI’s structured format and high sensitivity may identify previously undiagnosed or borderline cases, consistent with comparisons to longer diagnostic interviews such as the SCID. This reflects the instrument’s design, which prioritizes sensitivity and tolerates some false positives that can be clarified by clinical evaluation, rather than missing true cases. Additionally, for some disorders, the MINI considers a history of previous mental-health problems, resulting in a cumulative lifetime prevalence that can exceed the prevalence of current disorders [25]. Together, these factors likely contributed to the elevated prevalence observed in the control group. Among the MINI results, depression was the most commonly fulfilled criterion, followed by anxiety spectrum disorders. Notably, many participants reported a history of these issues rather than ongoing problems.

We also assessed PCOS-related features, including menstrual patterns, hirsutism, testosterone levels, and FAI. Although these characteristics differed significantly between the women with obesity with and without PCOS, we found no connection between these factors and sexual function. The only MFSQ item that differed between the obese groups was that the women with PCOS scored better on lubrication. The underlying cause of this is unclear, but it is possible that the elevated testosterone levels in PCOS positively influence lubrication. However, the evidence for this is conflicting [2, 5]. Previous studies have shown that elevated testosterone levels can be associated with increased sexual interest, satisfaction, orgasm, desire, and arousal [2, 37, 38]. However, other studies report a negative correlation between testosterone and sexual function, including reduced satisfaction, impaired orgasm, and dyspareunia [5, 6, 39]. Stovall et al. found links to improved sexual function only in individuals with the highest testosterone levels, and so postulated that testosterone requires a threshold level to exert a positive effect. This may explain the discrepancy noted in our study [38]. Increased testosterone levels are also associated with clinical features such as hirsutism and acne, which can negatively impact sexual function by affecting body image and self-esteem. It is possible that testosterone has both positive and negative effects that may cancel one another out [3, 39, 40]. This could explain why in this study sexual function in women with obesity with PCOS did not significantly improve postoperatively, despite the observed improvement in PCOS symptoms.

### Strengths and Limitations

A notable strength of this study is that patient recruitment was not based on individuals seeking medical advice for sexual problems. Instead, participants were recruited through the Department of Surgery, using a standard clinical assessment to determine eligibility for metabolic bariatric surgery. This approach ensured that patients with presumed lower sexual function were not selectively included, allowing for a more representative sample. Another strength is the study design, which included three groups: women with and without obesity, and women with and without PCOS. This design enabled us to differentiate between the effects of obesity and PCOS on sexual function. To our knowledge, no other study investigating the effects of metabolic bariatric surgery has employed such a comprehensive approach. By comparing these distinct groups, we aimed to provide a clearer understanding of the independent and combined effects of obesity and PCOS, making our findings particularly valuable in this field.

A limitation of this study is that the participants underwent either GBP or SG, due to a shift in the preferred surgical method during the study period. Notably, all GBP cases were within the PCOS group. However, evidence indicates that postoperative sexual function is not affected by the surgical method [32]. Importantly, however, extensive evidence shows that SG and GBP produce comparable improvements in weight loss, insulin sensitivity, and metabolic outcomes [41, 42]. Furthermore, studies specifically examining PCOS report similar improvements in hyperandrogenism, menstrual regularity, and ovulatory function after both procedures [18, 43]. To address this, we adjusted for procedure type and conducted sensitivity analyses, both of which yielded consistent results. Although procedure type remains a potential confounder, the available evidence indicates that the endocrine and metabolic changes relevant to our outcomes are largely independent of the specific surgical procedure.

A potential limitation of this study is that control participants were recruited at a different time than the surgical cohorts. This temporal difference may introduce cohort effects, such as variations in social context, recruitment channels, or seasonal and temporal stressors, which could influence the comparability of the groups.

The small sample size, resulting from the high dropout rate in the obese groups, may have reduced the study’s statistical power and representation of the broader population. While the dropouts were random and unlikely to have caused bias, they may have weakened the study and limited the ability to draw reliable conclusions.

## Conclusions

This study confirms that sexual function in women with obesity tends to improve after metabolic bariatric surgery, and provides evidence of an association between BMI and sexual function in women with PCOS. However, no direct associations were identified between mental-health status, testosterone levels, and sexual function, suggesting that the underlying causes of sexual dysfunction are multifaceted, involving both physical and psychological factors.

These findings emphasize the complex nature of sexual dysfunction in this population, and the necessity for further research to unravel the interplay of contributing factors. Such efforts are crucial for optimizing treatments and improving the quality of life of these women.

## Data Availability

The datasets used and/or analysed during the current study are available from the corresponding author on reasonable request.

## Abbreviations

PCOS: Polycystic ovary syndrome
OP: Obesity PCOS
ONP: Obesity non–PCOS
MFSQ: McCoy Female Sexuality Questionnaire
MINI: Mini International Neuropsychiatric Interview
FAI: Free androgen index
BMI: Body mass index
PMDD: premenstrual dysphoric disorder
SG: Sleeve gastrectomy
GBP: Roux–en–Y gastric bypass surgery
SHBG: sex hormone–binding globulin
MFG: modified Ferriman–Gallwey
DSM: Diagnostic and Statistical Manual of Mental Disorders
ICD: International Classification of Diseases
ECLIA: electrochemiluminescence immunoassay
CI: confidence interval
IQR: interquartile ranges
